# Supplementary material for: Nontrivial nanostructure, stress relaxation mechanisms, and crystallography for pressure-induced Si-I → Si-II phase transformation
Source: Nat Commun. 2022 Feb 21;13:982. doi: 10.1038/s41467-022-28604-1 (PMC8861166; doi:10.1038/s41467-022-28604-1)
Supplement: Supplementary file 1 — Supplementary Information [file 41467_2022_28604_MOESM1_ESM.pdf]

Supplementary material

Novel nanostructure, stress relaxation mechanisms, and crystallography for pressure-induced Si-I → Si-II phase transformation

Hao Chen[1], Valery I. Levitas[2,3], Dmitry Popov[4], Nenad Velisavljevic[5]

[1] *Key Laboratory of Pressure Systems and Safety, Ministry of Education, School of Mechanical and Power Engineering, East China University of Science and Technology, Shanghai 200237, P.R. China*

[2] *Iowa State University, Departments of Aerospace Engineering and Mechanical Engineering, Ames, Iowa 50011, USA*

[3] *Ames Laboratory, Division of Materials Science and Engineering, Ames, IA, USA*

[4] *HPCAT, X-ray Science Division, Argonne National Laboratory, Lemont, Illinois, USA*

[5] *HPCAT and Physics Division, Lawrence Livermore National Laboratory, Livermore, California 94550, USA*

Supplementary Note 1: Application of the traditional crystallographic theory

Transformation deformation gradients for three tetragonal variants of Si-II in a cubic coordinate are $\mathbf{F}_t^1 = \{a; a; b\}$, $\mathbf{F}_t^2 = \{a; b; a\}$, $\mathbf{F}_t^3 = \{b; a; a\}$, where $a = 1.175$ and $b = 0.553$ [1], i.e., transformation strains are very large, including large transformation volumetric strain $det\mathbf{F}_t = -0.237$. Using the crystallographic theory of martensite [2], we obtain that all variants are in a twin relationship with (110) twinning plane and very large twinning shear $\gamma = 1.6548$. Compatibility condition for twinned Si-II – Si-I coherent interface with normal $\mathbf{m}$ is

$$\mathbf{Q}' \cdot (\lambda \mathbf{Q} \cdot \mathbf{F}_t^2 + (1-\lambda)\mathbf{F}_t^1) = \mathbf{I} + \mathbf{b} \otimes \mathbf{m} \quad (1)$$

where λ is the volume fraction of variant 2 in the two-variant mixture, **Q** and $\mathbf{Q}'$ are orthogonal tensors, **I** is the unit tensor, and **b** is a vector. The solution to Supplementary Eq. (1) is

$$\mathbf{m} = [0.3313 \quad -0.0961 \quad 0.3962]; \quad \mathbf{b} = [0.4099\ 0.2151 - 0.8864]; \quad \lambda=0.7386. \qquad (2)$$

These results are far away from the results of MD simulations λ=0.5, $\mathbf{m} = [1; 1; 1]$, which shows that the traditional crystallographic theory is not applicable, in particular, due to large elastic strains.

Supplementary Note 2: Crystallographic theory for elastically deformed crystals

Actual deformation gradients for martensitic variants $\mathbf{F}_1$, $\mathbf{F}_2$, and $\mathbf{F}_3$, and distorted Si-I $\mathbf{F}_4$, as well as for Si-I matrix away from the interfaces $\boldsymbol{F}_0$, with respect to unstressed Si-I, determined from MD simulations, in a coordinate system **X**: $[110]$, **Y**: $[\bar{1}10]$, **Z**: $[001]$ are

$$\mathbf{F}_1 = \begin{bmatrix} 0.8998 & 0.5631 & 0.0133 \\ 0.0046 & 0.7145 & 0.0361 \\ 0.0027 & 0.0300 & 0.9769 \end{bmatrix}; \quad \mathbf{F}_2 = \begin{bmatrix} 0.9023 & -0.5982 & -0.0090 \\ -0.0641 & 0.7119 & 0.0126 \\ 0.0030 & -0.0698 & 0.9676 \end{bmatrix}; \qquad (3)$$

$$\mathbf{F}_3 = \begin{bmatrix} 1.0187 & 0.0473 & -0.0023 \\ 0.0352 & 1.0538 & -0.1832 \\ -0.2028 & 0.2361 & 0.5124 \end{bmatrix}; \quad \mathbf{F}_4 = \begin{bmatrix} 0.7926 & 0.0154 & 0.1625 \\ 0.1069 & 1.0139 & -0.2713 \\ -0.2078 & 0.2393 & 0.8684 \end{bmatrix};$$

$$\mathbf{F}_0 = \begin{bmatrix} 0.9035 & 0 & 0 \\ 0 & 0.9035 & 0 \\ 0 & 0 & 0.9035 \end{bmatrix}. \qquad (4)$$

The best way to take into account the elastic deformation of the austenite Si-I is to consider deformed Si-I as the new reference configuration. Then, relative deformation gradients are $\mathbf{F}_{i0} = \mathbf{F}_i \cdot \mathbf{F}_0^{-1}$, in particular,

$$\mathbf{F}_{10} = \begin{bmatrix} 0.9959 & 0.6232 & 0.0147 \\ 0.0051 & 0.7908 & 0.0400 \\ 0.0030 & 0.0332 & 1.0812 \end{bmatrix}; \quad \mathbf{F}_{20} = \begin{bmatrix} 0.9987 & -0.6621 & -0.010 \\ 0.071 & 0.7879 & 0.014 \\ 0.0033 & -0.0772 & 1.0709 \end{bmatrix}. \qquad (5)$$

Compatibility conditions for coherent interfaces between twins and twinned Si-II and Si-I with the normals $\mathbf{n}$ and $\mathbf{m}$ are:

$$\mathbf{Q}\cdot\mathbf{F}_{20}-\mathbf{F}_{10}=\mathbf{a}\otimes\mathbf{n}\,; \qquad \mathbf{Q}'\cdot(\lambda\mathbf{Q}\cdot\mathbf{F}_{20}+(1-\lambda)\mathbf{F}_{10})=\mathbf{I}+\mathbf{b}\otimes\mathbf{m}\,. \tag{6}$$

According to MD simulations, we use $\mathbf{n}$ = {111} and λ=0.5. Then the solution to Supplementary Eq. (6) is

$$\mathbf{m}=[-0.3370 \quad 0.2862 \quad -0.3899];\ \ \mathbf{b}=[-0.9050 \quad 0.1064 \quad 0.6937]. \tag{7}$$

The angle between $\mathbf{m}$ and $[11\bar{1}]$ is 25.15^{o}, which is quite large. This shows that even when elastic deformations of both Si-I and Si-II are taken into account, the traditional solution for twinned Si-II and Si-I interface is not an energy minimizer since MD simulations suggest a new, more sophisticated nanostructure.

Supplementary Note 3: Crystallographic analysis of the two-band nanostructure obtained in MD simulations

To determine deviation from the Hadamard compatibility conditions for bands I and II across {111} interface, evaluated in terms of deformation gradients averaged of each band, we introduce the new Cartesian coordinate system, with axes 1 along [111] direction, and mutually orthogonal axes 2 and 3 within {111} interface. For example, we the following orthonormal vector basis

$$\mathbf{a}_1=\begin{bmatrix}\frac{1}{\sqrt{3}} & \frac{1}{\sqrt{3}} & \frac{1}{\sqrt{3}}\end{bmatrix}^T,\ \mathbf{a}_2=\begin{bmatrix}-\frac{2}{\sqrt{6}} & \frac{1}{\sqrt{6}} & \frac{1}{\sqrt{6}}\end{bmatrix}^T,\ \mathbf{a}_3=\begin{bmatrix}0 & \frac{1}{\sqrt{2}} & -\frac{1}{\sqrt{2}}\end{bmatrix}^T. \tag{8}$$

Then, the orthogonal matrix transforming the original coordinate system to Supplementary Eq. (8) is $\mathbf{R}=\ [\mathbf{a}_1 \quad \mathbf{a}_2 \quad \mathbf{a}_3]$. The deformation gradients matrices in the current coordinate system, $\mathbf{F}_{\mathrm{iR}}=\mathbf{R}\cdot\mathbf{F}_{\mathrm{i}}\cdot\mathbf{R}^{\mathrm{T}}$, are as follows:

$$\mathbf{F}_{1\mathrm{R}} = \begin{bmatrix} 0.5087 & 0.1769 & 0.2078 \\ -0.2188 & 1.0669 & 0.0583 \\ -0.1864 & 0.0705 & 1.0156 \end{bmatrix}; \quad \mathbf{F}_{2\mathrm{R}} = \begin{bmatrix} 1.0876 & -0.0583 & -0.0363 \\ 0.3719 & 0.7281 & -0.2394 \\ 0.2889 & -0.2016 & 0.7660 \end{bmatrix};$$

$$\mathbf{F}_{3\mathrm{R}} = \begin{bmatrix} 1.0032 & 0.0877 & -0.1220 \\ -0.2448 & 0.7223 & 0.3177 \\ 0.1934 & 0.2393 & 0.8594 \end{bmatrix}; \quad \mathbf{F}_{4\mathrm{R}} = \begin{bmatrix} 0.8825 & 0.1025 & -0.3435 \\ -0.2789 & 0.8685 & 0.0240 \\ 0.1671 & 0.0316 & 0.9239 \end{bmatrix}, \tag{9}$$

while $\boldsymbol{F}_0$ does not change. Averaged over bands I , II and I+II deformation gradients are

$$\mathbf{F}^{\mathrm{I}} = c_1\mathbf{F}_{1\mathrm{R}} + c_2\mathbf{F}_{2\mathrm{R}} = \begin{bmatrix} 0.7981 & 0.0593 & 0.0857 \\ 0.0765 & 0.8975 & -0.0905 \\ 0.0513 & -0.0656 & 0.8908 \end{bmatrix}; \quad det\mathbf{F}^{\mathrm{I}} = 0.6246;$$

$$\mathbf{F}^{\mathrm{II}} = c_3\mathbf{F}_{3\mathrm{R}} + c_4\mathbf{F}_{4\mathrm{R}} = \begin{bmatrix} 0.9558 & 0.0935 & -0.2090 \\ -0.2582 & 0.7798 & 0.2023 \\ 0.1831 & 0.1577 & 0.8847 \end{bmatrix}; \quad det\mathbf{F}^{\mathrm{II}} = 0.6921;$$

$$\mathbf{F}^{\mathrm{av}} = \lambda_1\mathbf{F}_{1\mathrm{R}} + \lambda_2\mathbf{F}_{2\mathrm{R}} + \lambda_3\mathbf{F}_{3\mathrm{R}} + \lambda_4\mathbf{F}_{4\mathrm{R}} = \begin{bmatrix} 0.8712 & 0.0712 & 0.0043 \\ -0.0167 & 0.8952 & -0.0098 \\ 0.0908 & -0.0039 & 0.9203 \end{bmatrix};$$

$$det\mathbf{F}^{\mathrm{av}} = 0.718, \tag{10}$$

with

$c_1 = 0.5, c_2 = 0.5; c_3 = 0.6071, c_4 = 0.3929;$

$\lambda_1 = 0.3742, \lambda_2 = 0.3742, \lambda_3 = 0.1740, \lambda_4 = 0.1126.$

Also, $det\ \mathbf{F}_0 = 0.763$.

Let us evaluate jump in deformation gradient at different interfaces:

$$\mathbf{F}^{\mathrm{I}} - \mathbf{F}_0 = \begin{bmatrix} -0.1054 & 0.0593 & 0.0857 \\ 0.0765 & -0.0060 & -0.0905 \\ 0.0513 & -0.0656 & -0.0127 \end{bmatrix};$$

$$\mathbf{F}^{\mathrm{II}} - \mathbf{F}_0 = \begin{bmatrix} 0.0523 & 0.0935 & -0.2090 \\ -0.2582 & -0.1237 & 0.2023 \\ 0.1831 & 0.1577 & -0.0188 \end{bmatrix};$$

$$\mathbf{F}^{\mathrm{av}} - \mathbf{F}_0 = \begin{bmatrix} -0.0323 & 0.0712 & 0.0043 \\ -0.0167 & -0.0083 & -0.0098 \\ 0.0908 & -0.0039 & 0.0168 \end{bmatrix};$$

$$\mathbf{F}^{\mathrm{II}} - \mathbf{F}^{\mathrm{I}} = \begin{bmatrix} -0.1576 & -0.0342 & 0.2948 \\ 0.3348 & 0.1177 & -0.2928 \\ -0.1318 & -0.2232 & 0.0061 \end{bmatrix}. \qquad (11)$$

Five components of these matrices located in the first row and column characterize strains normal to {111} interface, which do not participate in the Hadamard compatibility conditions. The remaining 2×2 minors of these matrices describe interface distortion:

$$\mathbf{F}_{||}^{I} - \mathbf{F}_{0||} = \begin{bmatrix} -0.0060 & -0.0905 \\ -0.0656 & -0.0127 \end{bmatrix};$$

$$\mathbf{F}_{||}^{II} - \mathbf{F}_{0||} = \begin{bmatrix} -0.1237 & 0.2023 \\ 0.1577 & -0.0188 \end{bmatrix};$$

$$\mathbf{F}^{av} - \mathbf{F}_{0||} = \begin{bmatrix} -0.0083 & -0.0098 \\ -0.0039 & 0.0168 \end{bmatrix};$$

$$\mathbf{F}_{||}^{II} - \mathbf{F}_{||}^{I} = \begin{bmatrix} 0.1177 & -0.2928 \\ -0.2232 & 0.0061 \end{bmatrix}. \qquad (12)$$

For compatible interfaces that satisfy the Hadamard compatibility conditions, all matrices in Supplementary Eq. (12) are equal to zero. Since distortions are finite, to separate rotations and stretches, we

(a) add to all of them **I**, transforming them to deformation gradient at the interface $\mathbf{F}_{int}$,

(b) calculate the corresponding right stretch tensor $\mathbf{U}_{int} = (\mathbf{F}_{int}^T \cdot \mathbf{F}_{int})^{0.5}$,

(c) calculate the strain tensor $\mathbf{U}_{int}$-**I** and its two-principle value, which we will call incompatibility **Inc**.

Thus, for the band I-Si-I interface, **Inc**= (0.069; 0.087), i.e., corresponding strains are finite, which along with local stresses due to alternating twin tips cause nucleation of the band II. For the band II-Si-I interface, **Inc**= (0.117; 0.258), which causes much higher local stresses. For the band I-band II interface, **Inc**= (0.326; 0.201), which leads to huge local stresses. All these interfacial stresses are short-range and partially relax by local atomic rearrangements and loss of coherence; see interfaces in Fig. 2b. However, averaged over the band I and band II deformation gradient $\mathbf{F}_{av}$ produces with Si-I very small incompatibility **Inc**= (0.004; 0.021). In addition, normal to the {111} interface strain for bands I + II with res

pect to Si-I is also very small (-0.032) and difference in volumetric deformation gradients $det\, \mathbf{F}_{av}$ - $det\, \mathbf{F}_0$= -0.045, i.e., very small as well. All these facts produce almost self-accommodated band I + band II microstructure with small strain within Si-I matrix, and consequently, small long-range internal stresses.

Supplementary Note 4: The later stages of the evolution of Si-II structure under high pressure

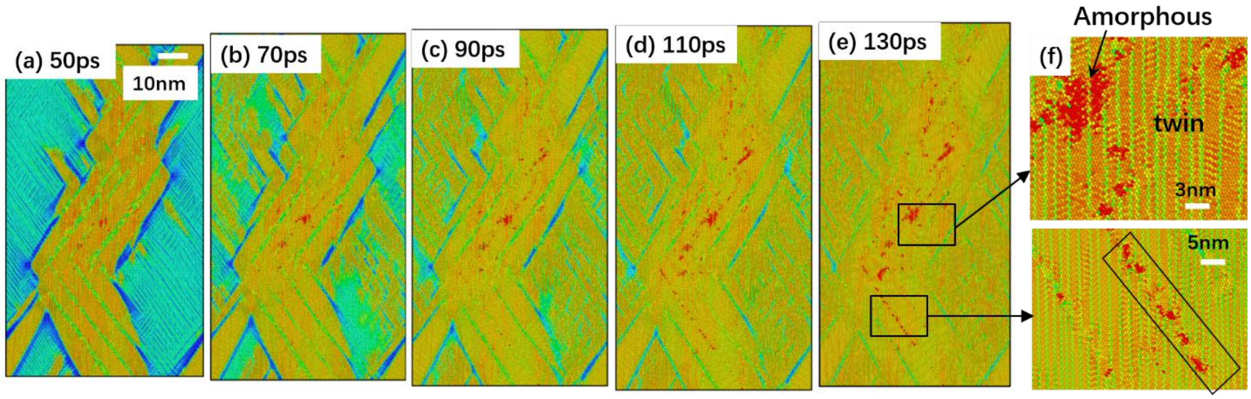

Supplementary Figure 1 The later evolution of the microstructure growing from two dislocation-induced nuclei. Bands II (green) gradually disappear during the evolution (a-e), and the final microstructure consists of twinned Si-II variants with deformation gradients $\mathbf{F}_1$ and $\mathbf{F}_2$, separated by (110) twinning plane (f). Twinned bands nucleated from different dislocation sites meet with each other and form an intermediate amorphous phase (f).

The initial evolution of Si-II exhibiting the nontrivial two-band structure is shown in Fig. 3 in the main text. The later evolution of the microstructure is shown in Supplementary Figure 1. With reducing volume fraction of remaining Si-I matrix and the elastic constraint due to the matrix, the width of band II (green region) and the volume fraction λ_3 of the variant $\mathbf{F}_3$ reduce. After Si-I matrix disappeared, band II disappears as well, leaving twinned Si-II and relaxing elastic stresses. Significant relaxation of lattice distortions in Si-II after completing PT was observed with XRD as well, indicated by "streaky" Laue and arch-like monochromatic beam reflections (Fig. 2). The final stable structure is a twinned structure consisted of $\mathbf{F}_1$ and $\mathbf{F}_2$, which corresponds to experiments (Fig. 2). Different twinned bands I nucleated from different dislocation sites can meet each other during evolution. When the twins match each other, perfect coalescence occurs, as

shown in Supplementary Figure 1(f). However, if they do not match each other, an intermediate amorphous phase is formed (Supplementary Figure 1).

We need to mention some discrepancies between MD simulation and experiment related, most probably to dislocational relaxation of internal stresses in experiment and absence of dislocation activity in MD simulations, due to chosen interatomic potential and smaller time and space scales. Thus, large elastic distortions from cubic Si-I and tetragonal Si-II observed in MD, relaxed in the experiment due to dislocation activity and arrest the nanostructure of the type shown in Fig. 3, leaving significant misorientation of different domains of Si-II. In contrast, in MD simulations, after completing PT, intermediate bands and misorientation disappear, leaving almost perfect twinned structure (Supplementary Figure 1).

Supplementary Note 5: High-pressure Laue diffraction

Supplementary Note 5.1: Experimental details and data analysis

The Si sample was put into a diamond anvil cell (DAC) with culets having 500μm diameters using a micromanipulator [3]. Re gasket was pre-indented down to 60μm and, after that, a hole of 300μm diameter was drilled in the gasket with a laser drilling machine [4]. The DAC was loaded by He as a pressure transmitting medium. While it is mentioned in [5] that He penetrates into SiO_2 glass and essentially changes its compressibility and is detected in Raman spectra, we did not find any mentioning of such effects for Si-I in the huge existing literature. Even in [5], neither GeO_2 glass nor crystalline phases of SiO_2 demonstrated this phenomenon.

Since pressure for Si-I to Si-II PT is well known and PT occurs at about 13 GPa ([3,6-10], we did not focus on precise detection of PT pressure in this study and did not use online Ruby fluorescence system to monitor pressure. The pressure was increased to approach the PT with the membrane system while measuring pressure using the off-line Ruby fluorescence system [11], available in

the experimental hutch. At 12.3 GPa, the DAC was mounted on the setup, and membrane pressure was further increased remotely in small steps, simultaneously collecting series of 2D scans. After each step of pressure increase, microstructural changes of the sample were watched in real time with ImageJ software [12]. Only if no notable changes were observed during multiple 2D scans was pressure increased by another step. After about 14 hours, the first changes in the sample due to the PT transition were observed, and membrane pressure was not increased any more. The transition was mainly completed in 38 minutes, although a small piece of the initial Si-I sample existed even more than 4 hours after the transition in the other parts of the studied area was finished (see below). The pressure was measured again with the Ruby system, 22 hours after the data collection procedure started, and found to be 13.8 GPa. Therefore, the pressure rate was less than 0.1 GPa/hour in average; although it was higher before the transition (as membrane pressure was increased) and, most likely, pressure still slightly increased also after the transition (as typically DACs have some pressure drifts even without an increase of membrane pressure). Note that the current pressure controlling system cannot control pressure with better precision than a few kbar/hour. Thus, PT occurred at ~13 GPa, and the estimated pressure increase during 38 minutes between initiation and completion of PT should not exceed 0.1 GPa. The pressure range and pressure rate here are like that in [3,6] (0.2-0.3 GPa/hour); thus, combining results from both experiments is legitimate.

As the sample position may slightly shift due to increased pressure, the sample was periodically re-centered on the rotation axis by doing absorption scans with a photodiode to keep the sample at the same position with respect to the X-ray beam and area detector [13,14]. No re-centering was done after alterations of the sample started, but after the data collection was finished, sample position with respect to the beam was re-determined, and shift of the sample relative to the

previously determined position was less than 1 μm. This indicates that all the 2D scans were collected on the same area of the sample across the transition.

The data analysis procedure consisted of two steps [3]. Laue reflections from the sample were indexed first in order to identify crystals and find their orientations. After that, maps of reflections have been generated to detect changes of crystal morphology and deformation of the crystals due to the transition. Indexation and mapping of reflections were done with software polyLaue developed in-house by D. Popov [3]. Diffraction patterns have been visualized with the Dioptas program [15,16]. Maps of reflections have been visualized with the Fit2d program [17,18]. The strongest reflections from diamonds may damage the area detector. Therefore, such reflections were blocked by a detector mask [3]. All other reflections from diamonds have been identified by indexation and excluded from further data analysis.

Supplementary Note 5.2: Results and discussion

A typical diffraction pattern and map of a reflection right on the onset of the transition are presented in Supplementary Figure 2. The numbering of all other 2D scans considered below starts from this scan. During the first five 2D scans, starting from the onset, the sample exhibited severe deformation. Positions of reflections substantially variated across the sample, indicating variation of crystal orientation. At the same time, crystal morphology also changed because some parts of the sample transformed to Si-II. All the maps of reflections obtained during scans 1-5 were very different from each other, even if they were obtained from adjacent scans because sample alterations took shorter periods of time than required to collect one 2D scan. Therefore, changes of the sample during scans 1-5 could not be identified.

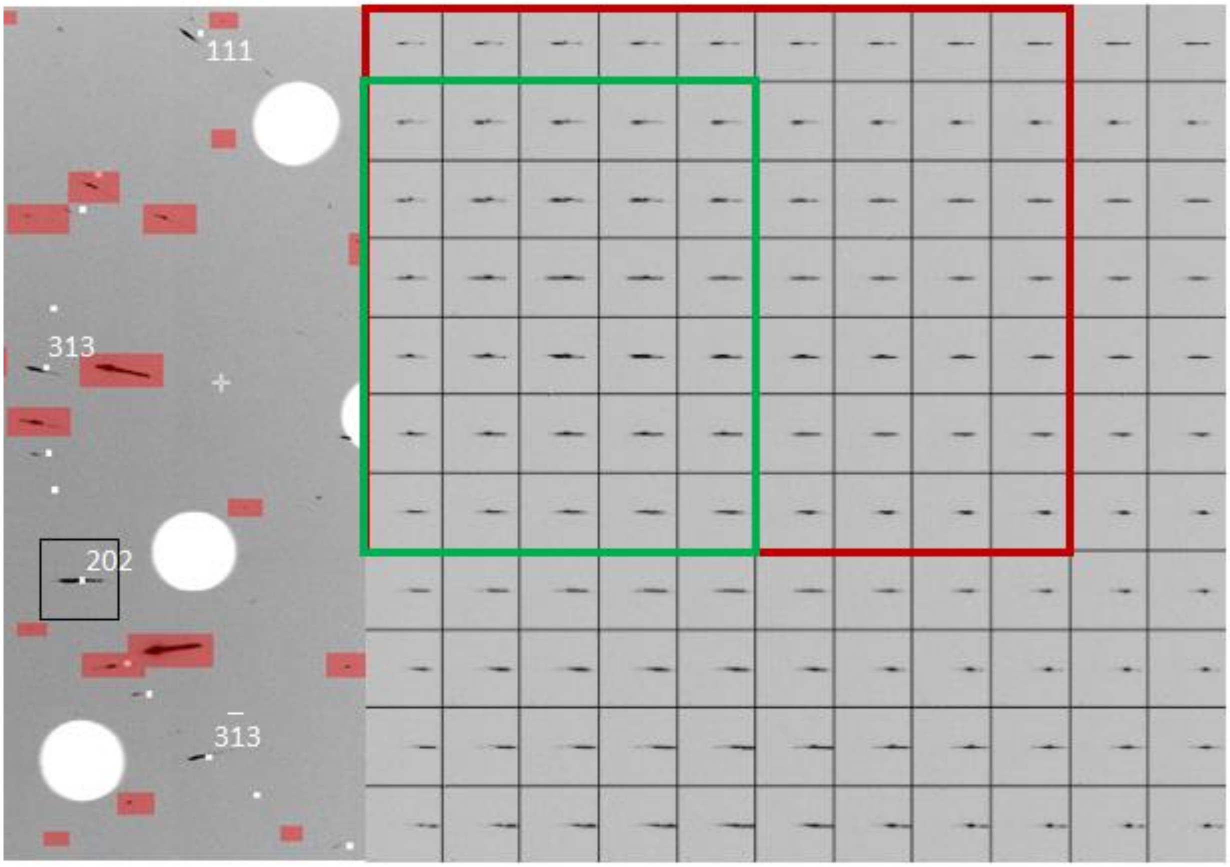

Supplementary Figure 2 Laue diffraction pattern (left) and map of 202 reflection (right) from Si-I obtained right on the onset of the $\alpha \rightarrow \beta$ transition. Predicted positions of reflections, assuming the highest X-ray energy limit of 80keV and d-values of reflections larger than 0.5Å, are shown on the diffraction pattern as white rectangles slightly shifted to the right not to overlap with the observed reflections. Black rectangle in the diffraction image denotes an area used to build the composite frame in the right. The step size of the translational scan was 1 μm. The numbering and time intervals of the scans mentioned in the Supplementary materials, movies and other figures start from the scan presented in this figure. Areas within red and green boxes are occupied by the Si-I crystals referenced in the Supplementary materials as crystals 1 and 2, respectively. These areas are presented in Supplementary movies 1-4. Reflections from diamonds on the Laue pattern are denoted by red transparent rectangles. White circles on the Laue image are due to the detector mask.

Starting from 2D scan 6, only a few pieces of the original Si-I sample remained in the studied area. Still, these crystals were stable enough to produce maps of reflections reproducible during multiple 2D scans, and, therefore, it was possible to identify these crystals. Maps of reflections and diffraction patterns, obtained during multiple 2D scans from two of these crystals, were combined in the same order as these maps, and diffraction patterns were obtained (Supplementary movies 1-4). These crystals are referenced below as crystal 1 (Supplementary movies 1 and 2, area outlined in red in Supplementary Figure 2) and crystal 2 (Supplementary movies 3 and 4, area outlined in green in Supplementary Figure 2).

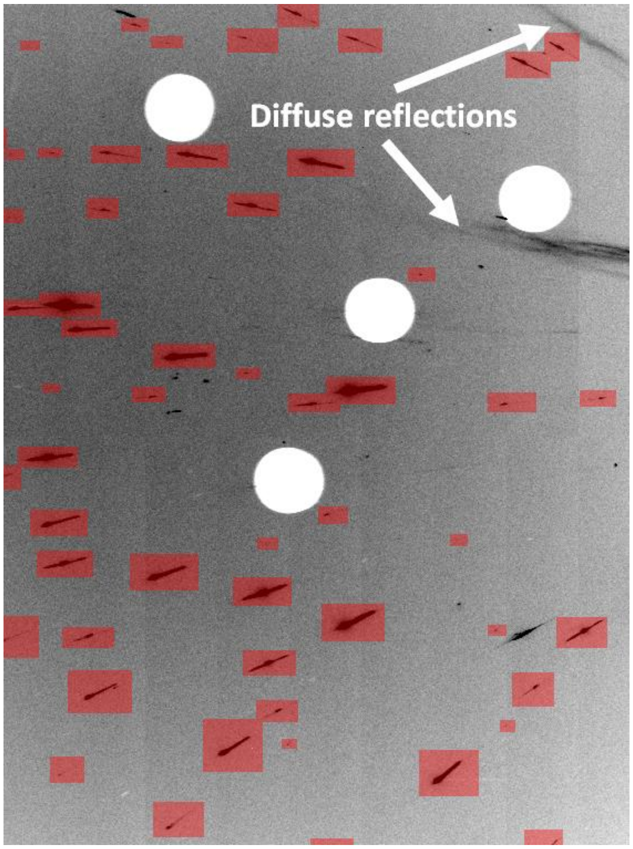

Supplementary Figure 3 Diffuse reflections from Si-II. White circles are due to the detector mask. Red rectangles denote reflections from diamonds.

After the onset of the transition, very diffuse reflections from the high-pressure phase were observed (Supplementary Figure 3). 'Streaky' shape of these reflections indicated that the product Si-II phase exhibited strong misorientation of its nanodomains. This observation agrees with the previous results [3,6]. Indexation of reflections from Si-II was impossible.

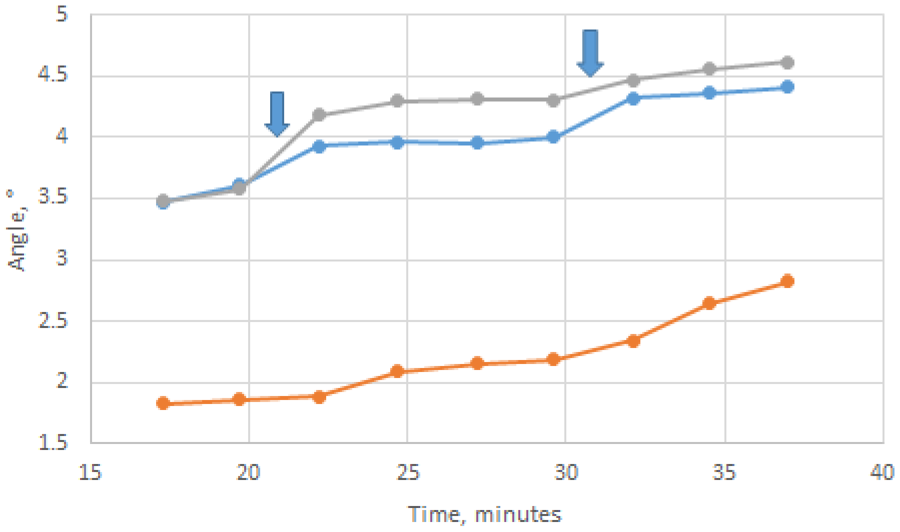

Supplementary Figure 4 Angular deviations versus time of Si-I crystals, referenced in the Supplementary materials as crystals 1 (gray) and 2 (blue), from the parental Si-I crystal at the onset of the $\alpha \rightarrow \beta$ transition. These deviations may include rigid-body rotation of the entire sample due to a change in its shape. Angles between crystals 1 and 2 versus time (yellow). Vertical arrows denote time points when crystal 1 exhibited a much faster decrease of its size than on average. Time starts from the scan presented in Supplementary Figure 2.

Due to the deformation process, orientations of crystals 1 and 2 were shifted by about 3.5° with respect to the sample orientation, which was observed right on the onset of transition. Orientations of crystals 1 and 2 were also shifted with respect to one another by about 1.8°. Absolute and relative angular deviations of crystals 1 and 2 gradually increased further across the transition (Supplementary Figure 4). In about 20 minutes, absolute and relative angular shifts

reached about 4.5° and 2.8°, respectively. Strong deformation of the sample right after the onset of the transition and relative angular shifts of crystals 1 and 2 are caused by heterogeneous internal stresses due to transformation strain in Si- II phase. Interaction of the sample with pressure medium, diamond, and gasket mainly contributes to the absolute angular shifts due to rigid-body rotation of the entire sample but essentially does not affect relative angular shifts of crystals 1 and 2.

Results from crystal 1 indicate that the speed of the transition can be fundamentally faster comparing to what can be concluded based on the total time of transition. Starting from 2D scan 6, crystal 1 existed for about 26 minutes before it was completely transformed to Si-II. However, mostly, the transformation took place in two short periods of time, less than a minute each, between scans 9 and 10 and between scans 13 and 14 (Supplementary movies 1,2). Interestingly, during these two periods of time, both crystals 1 and 2 exhibited shifts of their absolute orientations notably higher than on average while their relative orientation changed as normal (Supplementary Figure 4 and 5). This is the indication that these two periods of time, when the transition was fundamentally faster than in average, were accompanied by substantial changes of orientation of the entire sample or, if the sample was fragmented (broken) to smaller pieces because of the deformation, by reorientation of the pieces of the sample containing crystals 1 and 2 as a whole.

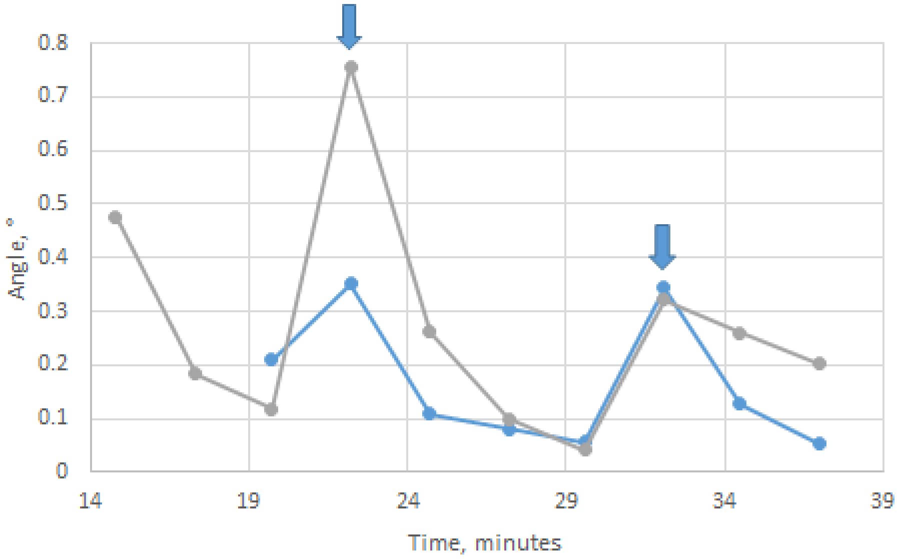

Supplementary Figure 5 Angular shifts between adjacent scans versus time for Si-I crystals, referenced in the Supplementary materials as crystals 1 (gray) and 2 (blue), across the $\alpha \rightarrow \beta$ transition in Si. Vertical arrows denote time points when crystal 1 exhibited a much faster decrease of its size than on average. Time starts from the scan presented in Supplementary Figure 2.

Variations of the transition speed can be explained by the known fact that the speed of transition is substantially controlled by crystal lattice defects (not included in the simulations), introducing activation barriers. Due to heterogeneity of the defects distribution, interface propagation rate may vary significantly. After waiting for a long time near an obstacle with high activation barrier and then overcoming it with the help of strong thermal fluctuation, some portion of the interface may travel fast for a relatively large distance until it meets the next strong barrier. At the same time, crystal 2 does not exhibit any notable acceleration of the transition simultaneously with crystal 1 (Supplementary movies 1-4). This indicates that crystal lattice defects, individual for each of the crystals, are the major factor defining the speed of transition. Starting from 2D scan number 16, after 38 minutes from the first 2D scan, no Laue reflections from Si-I except those from crystal 2 were observed on the studied area of the sample. Crystal 2

was not transformed to Si-II phase even 4 hours 2 minutes after scan 16, when Laue diffraction measurements were finished (Supplementary movies 3, 4). This agrees with the defect-controlled transformation speed and thermally activated mechanism of the interface motion.

Crystals of Si-I, coexisting with Si-II, may exhibit changes in their deformation state, which agrees with the previous results [3,6]. A possible explanation of these changes is essentially the same as for the reorientations of Si-I crystals across the transition. For instance, crystal 2 produced sharper reflections starting from 2D scan 10, compared to the previous scans, indicating a relaxation process. When the transition was almost finished in the studied area and only crystal 2 coexisted with the high-pressure phase, crystal 2 exhibited quite notable changes in its deformation state. Reflection 111 became much more broaden comparing to $1\bar{1}1$. One possible explanation of this observation is that crystal 2 may become slightly bent cylindrically around a direction close to $1\bar{1}1$ [19]. Similar lattice rotation of Si-I crystals coexisting with Si-II has been reported previously [3,6].

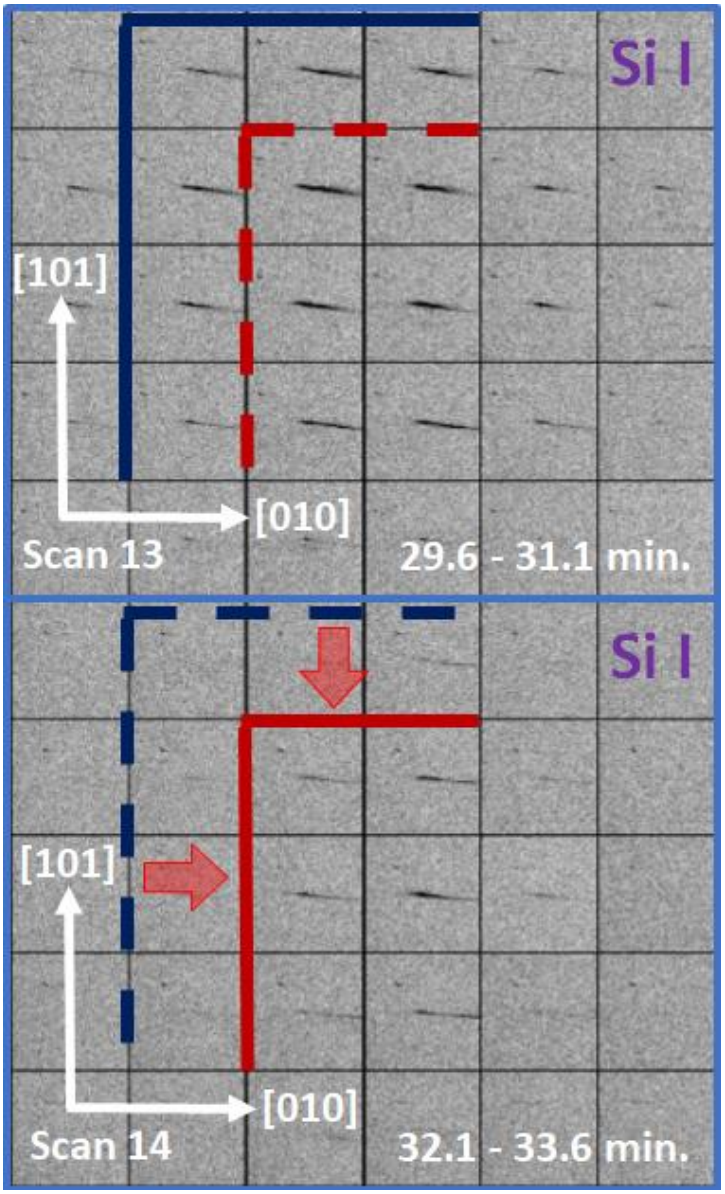

Supplementary Figure 6 Shifts of the Si-I / Si-II interfaces (denoted by red arrows), projected onto a {110} plane of the Si-I crystal referenced as crystal 1 in the Supplementary materials. Shifts of the Si-I / Si-II interfaces are detected from maps of 202 reflection. The intensity scaling of both maps is kept the same before and after the shift as defined in Fit2d program [17,18]. The interfaces before and after their shifts are denoted by dark blue and red lines, respectively; solid lines denote currently existing interfaces, and dashed lines denote interfaces existing in different states. The numbering and time intervals of the scans start from the scan presented in Supplementary Figure 1.

Maps of crystals 1 and 2, obtained in real-time, provide information on the orientation of the Si-I / Si-II interface as projected onto the {110} plane approximately perpendicular to the incident beam. Edges of crystals 1 and 2, projected onto the {110} plane, are oriented about parallel to one of the following directions <111>, <110> or <100> (Supplementary movies 1-4). However, this criterion for the interface is not reliable because the edges of the crystals may have been formed by cracking the original sample due to the deformation process and not by the interface. Much more reliable way to detect the interface between parental and product phases is the observation of shifts of the parental phase edges across the transition. Crystal 1 exhibited very notable shifts of the Si-I / Si-II interface, detected this way, between scans 9 and 10 (Supplementary movies 1, 2) and between scans 13 and 14 (Supplementary Figure 6 and movies 1 and 2). These shifts indicate interfaces parallel to <110> and <100>.

Experiments also reveal that: (a) this PT is rather nucleation controlled because after nucleation it proceeds until completing at the same pressure; (b) kinetics is thermally activated, with two burst-like fast transformation increments and slow transformation otherwise; (c) speed of transition is controlled by kinetic factors not included into the simulations. These factors are defined by defects of the original Si-I single-crystal because the speed of the PT is quite different in different regions of crystals.

Supplementary Note 6: Reconstructive versus martensitic phase transformation from Si-I to Si-II

The potential problem often arises from the different classifications of structural PTs in different communities. One of the communities, which focuses on bonding between atoms, calls PT a reconstructive if it involves breaking/changing the bonds; see the book [20]. Alternative PTs without bond breaking are often called displacive. Then the PT from the semiconductive Si-I to

metallic Si-II clearly belongs to reconstructive PTs [20-23]. There are some other features of these groups of PTs, e.g., reconstructive PT involves large displacements, and no group-subgroup relationship exists between phases, while for displacive PTs, displacements are small, and phases obey group-subgroup relationship [20]. Communities that are concerned with material science, crystallographic, and microstructural aspects do not care about bonding and divide structural PTs into diffusive and diffusionless or martensitic [2,24-27]. Diffusive PTs are accompanied by diffusion and exchange of atomic neighbors during PT, like amorphization and PT occurring via intermediate disordered/amorphous state, precipitation, eutectoid, and massive PT, as well as ordering. Martensitic PTs, in contrast, do not involve diffusion and exchange of atomic neighbors, and mapping of positions of two lattices can be presented by homogeneous deformation (transformation deformation gradient) and some additional intra-cell displacements called shuffles or shifts. Twinning is considered a particular case of martensitic PT, for which the same lattices are connected by a transformation deformation gradient representing simple shear along the twinning plane. Internal stresses and evolution of martensitic microstructure are determined by minimization of the elastic energy, which, in addition to an external load, is completely determined by the field of the transformation deformation gradient and is independent of shuffles. That is why the main parameter in the crystallographic theory and theory of the microstructure is the transformation deformation gradient [2,27], independent of breaking or not breaking bonds. These theories determine, based on the transformation deformation gradient and assumed mode of the lattice-invariant shear (slip or twinning), normal to the habit and twining planes, the volume fraction of twin-related martensitic variants (see Supplementary Eqs. (1)-(2)), and orientation relationship between lattices of different phases [2,27].

Most of the reconstructive PTs discussed in the monograph [20] are described by the crystallographic theory of martensitic PTs, including PT in iron [27], shape memory alloys [2], plutonium [28], and hexagonal and rhombohedral graphite to hexagonal and cubic diamond and similar PTs in BN [29,30]. It is mentioned in [30] that graphite-like phases of BN have very strong covalent bonds with sp^2 hybridization within the hexagonal planes and weak van der Waals bonds between these planes, while cubic or wurtzitic superhard BN have only covalent (partially ionic) bonds, with tetrahedral three-dimensional sp^3 hybridization; the same is true for graphite and diamond. Despite the reconstructive PT between these phases, all aspects of crystallographic and microstructure formation theory are applied in [29,30] to these PTs; they are called martensitic in [29].

With a full understanding of change in bonding during PT Si-I – Si-II in [1,31], it is treated as martensitic PT in which the transformation deformation gradient connects atoms of Si-I and Si-II lattice even without shuffles. All continuum theories of PTs involving the transformation deformation gradient (e.g., based on energy minimization [2] or phase field theories [32-31], including those for Si [32-34]) do not distinguish bonding. However, some works distinguish between reconstructive martensitic PTs, for which no group-subgroup relationship exists between phases, and "weak" martensitic PTs, for which phases obey group-subgroup relationship [35,36]. Reconstructive martensitic PTs are more complex for modeling because the reverse PT may occur to different variants of the austenite related by a lattice-invariant shear. But this is not the case for cubic to tetragonal PT Si-I – Si-II.

To summarize, various definitions of martensitic and reconstructive PTs are used by different research communities depending on their goals. For example, the firefighters distinguish a violin from a piano in a simple way: the piano burns longer, which is sufficient for their goals.

Since our main goal here is crystallography and microstructure rather than atomic bonding, we call Si-I to Si-II PT martensitic. Our results and conclusions would not change if we called them reconstructive or by any other name.

Supplementary References:

1. Zarkevich, N.A., Chen, H., Levitas, V.I. and Johnson, D.D., 2018. Lattice instability during solid-solid structural transformations under a general applied stress tensor: Example of Si-I→ Si-II with metallization. *Phys. Rev. Lett.* 121(16), p.165701.

2. Bhattacharya, K., 2003. Microstructure of Martensite. Why it Forms and How It Gives Rise to the Shape-Memory Effect. Oxford University Press.

3. Popov, D.; Velisavljevic, N. and Somayazulu, M. (2019) Mechanisms of Pressure-Induced Phase Transitions by Real-Time Laue Diffraction. *Crystals* 9, 672

4. Hrubiak, R.; Sinogeikin, S.; Rod, E.; Shen, G. The laser micro-machining system for diamond anvil cell experiments and general precision machining applications at the High Pressure Collaborative Access Team. *Rev. Sci. Instrum.* ***86***, 072202 (2015).

5. Shen, G.; Mei, Q.; Prakapenka, V.; Lazor, P.; Sinogeikin, S.; Meng, Y. and Park C. Effect of helium on structure and compression behavior of SiO2 glass. *PNAS* **108**, 6004-6007 (2011).

6. Popov, D., Park, C., Kenney-Benson, C. & Shen, G. High pressure Laue diffraction and its application to study microstructural changes during the $\alpha \rightarrow \beta$ phase transition in Si. Rev. Sci. Instrum. 86, 072204 (2015).

7. Jamieson, J. C. Crystal structures at high pressures of metallic modifications of silicon and germanium. Science 139, 762-4 (1963).

8. Hu, J. Z., Merkle, L. D., Menoni, C. S. & Spain, I. L. Crystal data for high-pressure phases of silicon. Phys. Rev. B 34, 4679 (1986).

9. McMahon, M. I., Nelmes, R. J., Wright, N. G., and Allan, D. R., Pressure dependence of the Imma phase of silicon. Phys. Rev. B 50(2), 739 (1994).

10. Shen, G., Ikuta, D., Sinogeikin, S., Li, Q., Zhang, Y., and Chen, C., Direct Observation of a Pressure-Induced Precursor Lattice in Silicon. Phys. Rev. Lett. 109, 205503 (2012).

11. Mao, H.K.; Xu, J.; Bell, P.M. Calibration of the Ruby pressure gauge to 800-kbar under quasi-hydrostatic conditions. *J. Geophys. Res.: Solid Earth* **1986**, *91***,** 4673-4676

12. https://imagej.nih.gov/ij/

13. Smith, J.S.; Desgreniers, S. Selected techniques in diamond anvil cell crystallography: centring samples using X-ray transmission and rocking powder samples to improve X-ray diffraction image quality. *J. Synchrotron Radiat.* **2009**, *16*, 83–96

14. Smith, J.S,; Rod, E.A.; Shen G. Fly scan apparatus for high pressure research using diamond anvil cells. *Rev. Sci. Instrum.* **2019**, *90*, 015116

15. Prescher, C.; Prakapenka, V. DIOPTAS: a program for reduction of two-dimensional X-ray diffraction data and data exploration. *High Pressure Res.* **2015**, 35, 223–230

16. http://www.clemensprescher.com/programs/dioptas

17. Hammersley, A. P.; Svensson, S. O.; Hanfland, M.; Fitch, A. N.; Hausermann, D. Two-dimensional detector software: from real detector to idealized image or two-theta scan. *High Pressure Res.* **1996**, *14***,** 235-248

18. http://www.esrf.eu/computing/scientific/FIT2D/

19. Yang W., Larson B. C., Ice G. E., Tischler J. Z., Budai J. D., and Chung K.-S. Spatially resolved Poisson strain and anticlastic curvature measurements in Si under large deflection bending. *Applied Physics Letters* **2003**, 82(22), 3856

20. P. Toledano and V. Dmitriev, Reconstructive Phase Transitions, (World Scientific, New Jersey, 1996).

21. A. Mujica, A. Rubio, A. Munoz, and R. J. Needs. High-pressure phases of group-IV, III–V, and II–VI compounds. Rev. Mod. Phys. 75, 863 (2003)
22. L. Q. Huston, A. Lugstein, J. S. Williams, and J. E. Bradby. The high pressure phase transformation behavior of silicon nanowires. APL 113, 123103 (2018)
23. E. E. McBride, A. Krygier, A. Ehnes, E. Galtier, M. Harmand, Z. Konôpková, H. J. Lee, H.-P. Liermann, B. Nagler, A. Pelka, M. Rödel, A. Schropp, R. F. Smith, C. Spindloe, D. Swift, F. Tavella, S. Toleikis, T. Tschentscher, J. S. Wark and A. Higginbotham. Phase transition lowering in dynamically compressed silicon. Nature Phys 15, 89–94 (2019)
24. Porter D, Easterling K. Phase transformation in metals and alloys. New York: Van Nostrand Reinhold; 1992.
25. Fultz B. Phase transitions in materials. Cambridge University Press, Cambridge, 2014.
26. J. Christian, G. Olson, M. Cohen. Classification of Displacive Transformations: What is a Martensitic Transformation? J. de Physique IV, Colloque C8, supplement au J. de Physique III, 1995, 5, pp. 3-10.
27. Wayman, C.M., 1964. Introduction to the Crystallography of Martensitic Transformation. New York, Macmillan.
28. Adler, P., Olson, G., Stevens, M., and Gallegos, G., 1992. On the Constitutive Relations for δ-α and α - δ Martensitic-Transformation Plasticity in Plutonium Alloys. Acta Metall. Mater 40, 1073-1082.
29. V. F. Britun and A. V. Kurdyumov. Analysis of the effect of nonhydrostatic compression conditions on direct phase transformations in carbon. Powder Metallurgy and Metal Ceramics, 41, 7-8, 2002.

30. L. C. Nistor , G. Van Tendeloo & G. Dinca (2005) Crystallographic aspects related to the high pressure–high temperature phase transformation of boron nitride, Philosophical Magazine, 85, 1145-1158.
31. Malyushitskaya, Z.V., 1999. Mechanisms Responsible for the Strain-Induced Formation of Metastable High-Pressure Si, Ge, and GaSb Phases with Distorted Tetrahedral Coordination. Inorganic Materials 35, 425-430.
32. Levitas, V. I. Phase field approach for stress- and temperature-induced phase transformations that satisfies lattice instability conditions. Part 1. General theory. Int. J. Plast. 106, 164-185 (2018).
33. Babaei, H. & Levitas, V. I. Phase field approach for stress- and temperature-induced phase transformations that satisfies lattice instability conditions. Part 2. Simulations of phase transformations Si-I↔Si-II. Int. J. Plast. 107, 223-245 (2018).
34. Babaei, H. & Levitas V. I. Finite-strain scale-free phase-field approach to multivariant martensitic phase transformations with stress-dependent effective thresholds. J. Mech. Phys. Solids 144, 104114 (2020).
35. Denoual, C., Vattre, A., 2016. A phase field approach with a reaction pathways-based potential to model reconstructive martensitic transformations with a large number of variants. J. Mech. Phys. Solids, 90, 91-107.
36. K. Bhattacharya, S. Conti, G. Zanzotto, J. Zimmer. Crystal symmetry and the reversibility of martensitic transformations. Nature, 428, 55-59, 2004.
