# Supplementary material for: Nontrivial nanostructure, stress relaxation mechanisms, and crystallography for pressure-induced Si-I → Si-II phase transformation
Source: Nat Commun. 2022 Feb 21;13:982. doi: 10.1038/s41467-022-28604-1 (PMC8861166; doi:10.1038/s41467-022-28604-1)
Supplement: Supplementary file 3 — Description of Additional Supplementary Files [file 41467_2022_28604_MOESM3_ESM.pdf]

Description of Additional Supplementary Files

File Name: Supplementary Movie 1
Description: Maps of 202 reflection (left top) and Laue diffraction patterns (right) from Si-I crystal, referenced as crystal 1 in supplementary materials, obtained across the $\alpha \rightarrow \beta$ transition, along with maps of a diffuse reflection from Si-II, collected on the same area of the sample (left bottom). Positions of diffraction spots have been predicted assuming the highest X-ray energy limit of 80keV and the smallest d-values of reflections gradually changing from 0.5Å to 0.9Å along with the data collection routine. Green and yellow rectangles in the diffraction images denote areas used to build the corresponding composite frames on the left. Green and yellow rectangles in the maps of reflections correspond to the position within the sample where the diffraction patterns on the right have been collected.

File Name: Supplementary Movie 2
Description: Maps of 313 and 111 reflections from Si-I crystal, referenced as crystal 1 in supplementary materials, obtained across the $\alpha \rightarrow \beta$ transition. Green rectangles correspond to the position within the sample where the diffraction patterns presented in movie 1 have been collected. Strong reflections from diamonds are present along with the 313 reflection.

File Name: Supplementary Movie 3
Description: Maps of $3\bar{1}3$ reflection (left) and Laue diffraction patterns (right) from Si-I crystal, referenced as crystal 2 in supplementary materials, obtained across the $\alpha \rightarrow \beta$ transition. Positions of diffraction spots have been predicted assuming the highest X-ray energy limit of 80keV and d-values of reflections larger than 0.6Å. Green rectangles in the diffraction images denote areas used to build the corresponding composite frames on the left. Green rectangles in the maps of reflections correspond to the position within the sample where the diffraction patterns on the right have been collected. Reflections from the different crystals are present on the maps until scan 14.

File Name: Supplementary Movie 4
Description: Maps of 111 and $1\bar{1}1$ reflections from Si-I crystal, referenced as crystal 2 in supplementary materials, obtained across the $\alpha \rightarrow \beta$ transition. Green rectangles correspond to the position within the sample where the diffraction patterns presented in movie 3 have been collected. Strong reflections from diamonds are present along with the $1\bar{1}1$ reflection. Reflections from different crystals are present until scan 14.

File Name: Supplementary Movie 5
Description: Series of oscillation diffraction patterns from Si-II obtained using a monochromatic beam with 1° angular step [14]. Red circles denote predicted diffraction lines of Si-II.

File Name: Supplementary Movie 6
Description: Evolution of the twinning microstructure from MD simulations.
